# Supplementary material for: Decoding the transcriptome of Duchenne muscular dystrophy to the single nuclei level reveals clinical-genetic correlations
Source: Cell Death Dis. 2023 Sep 7;14(9):596. doi: 10.1038/s41419-023-06103-5 (PMC10482944; doi:10.1038/s41419-023-06103-5)
Supplement: Supplementary file 1 — Supplemental material [file 41419_2023_6103_MOESM1_ESM.pdf]

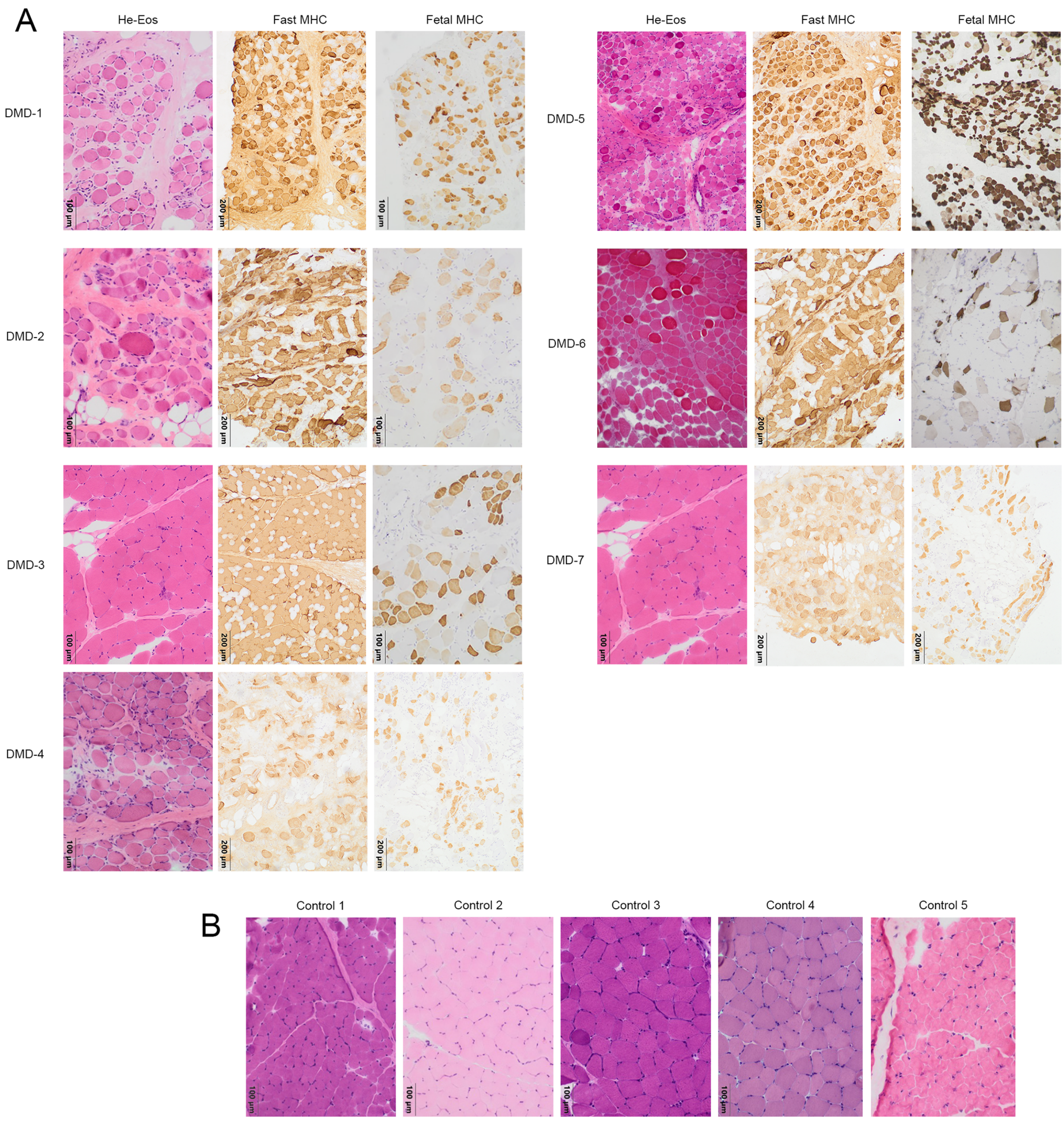

Supplemental Figure 1: Histological features of the muscle samples included in the study.

A) Hematoxilin-eosin staining (He-Eos), and immunostainings of fast myosin heavy chain (MHC) and fetal MHC in the seven DMD patients. He-Eos shows a variable dystrophic pattern among the different DMD patients and Fast MHC staining shows a predominance of fast fibers in DMD patients. An widespread regeneration process is observed by the presence of fetal MHC-positive myofibers in DMD samples. B) Hematoxilin-eosin staining in the five control muscle samples included in the study.

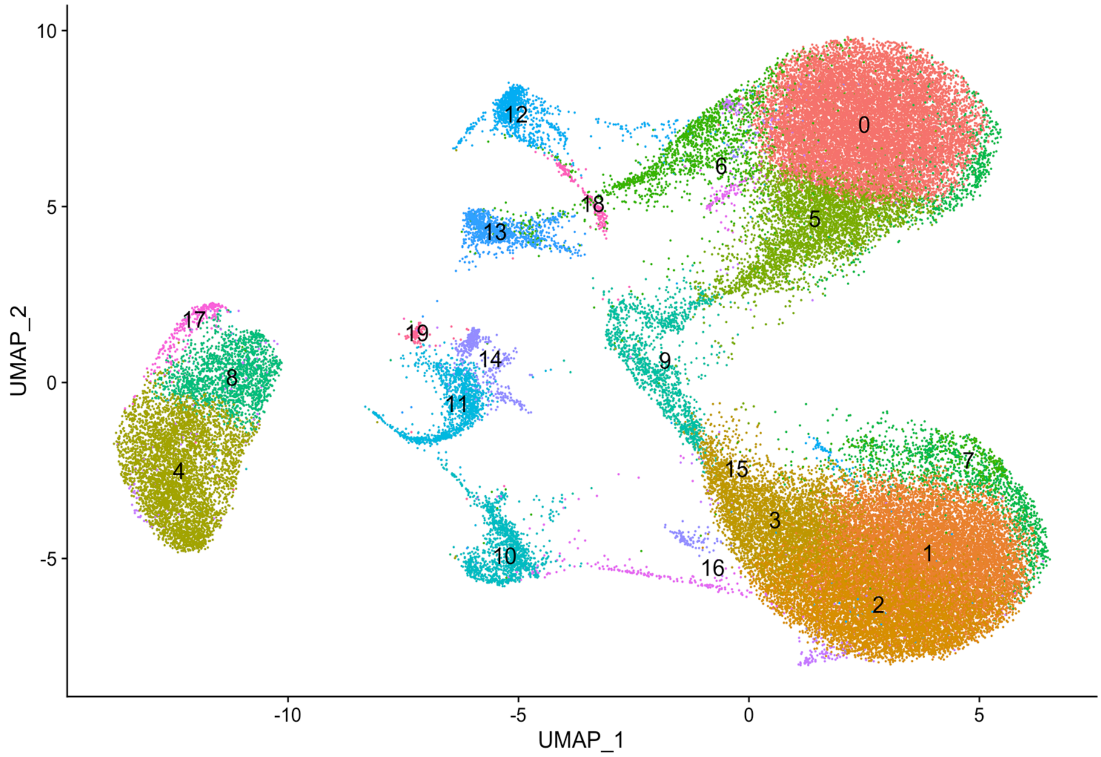

Supplemental Figure 2: UMAP visualization of all nuclei from control and DMD individuals coloured by cluster number before identities were obtained based on the expression of canonical markers.

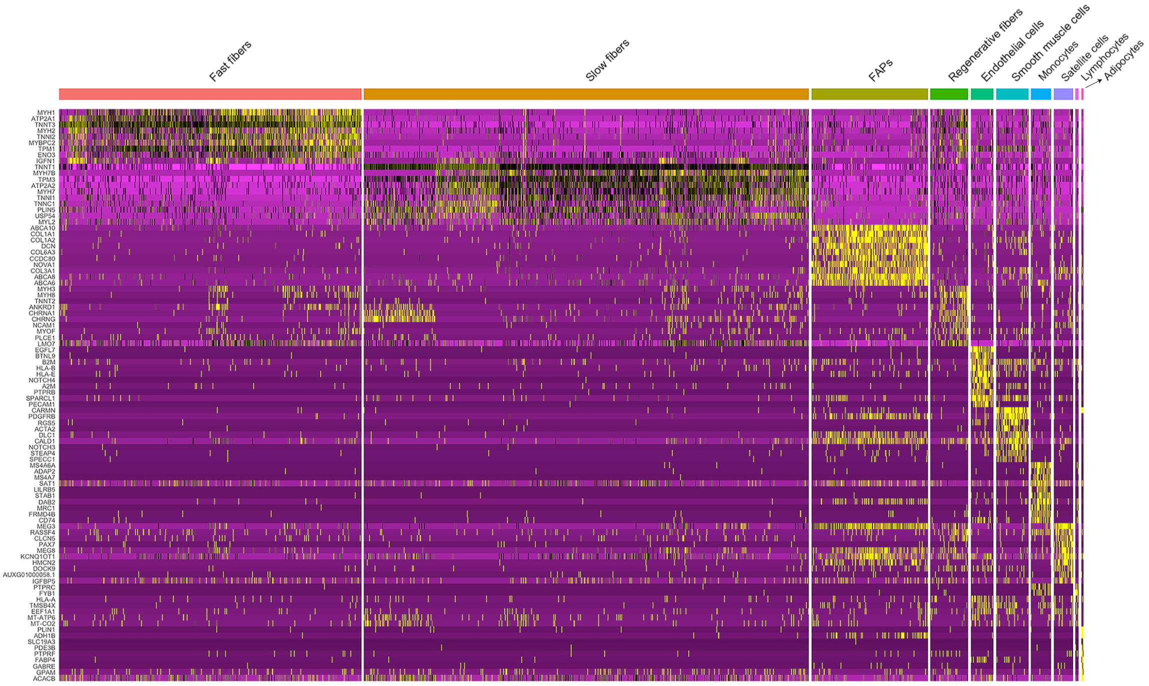

Supplemental Figure 3: Heatmap showing the 10 most upregulated genes expressed on each cell cluster.

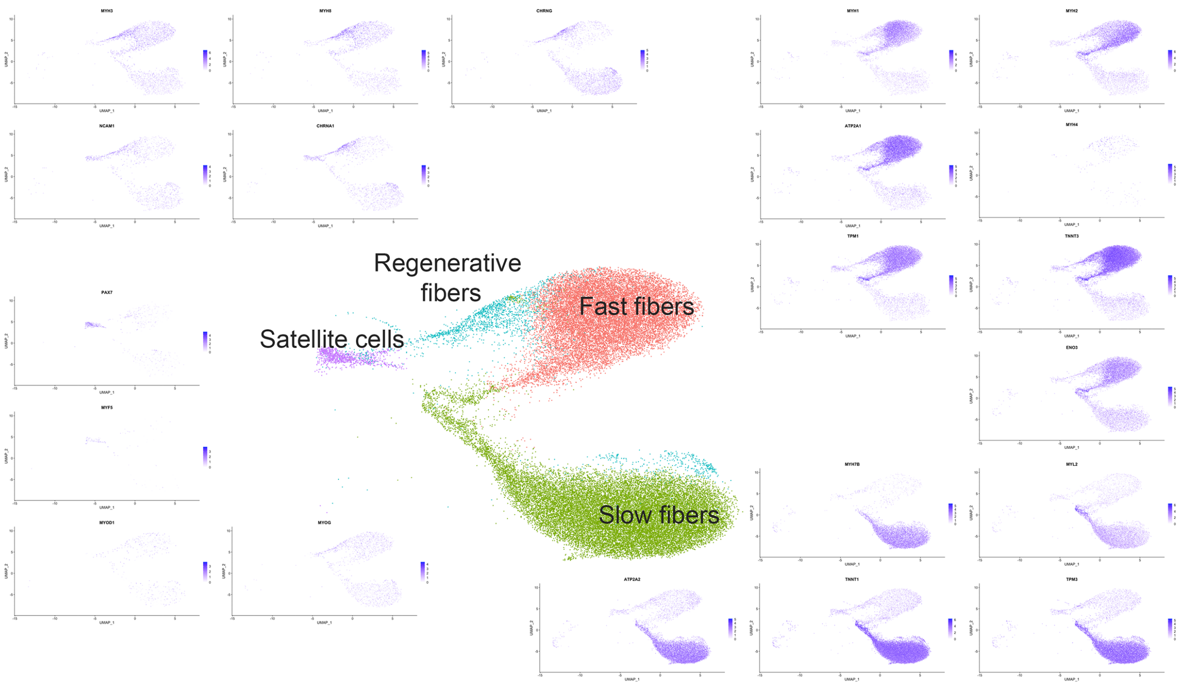

Supplemental Figure 4: Feature-plot showing expression of specific genes in clusters of myonuclei and satellite cells.

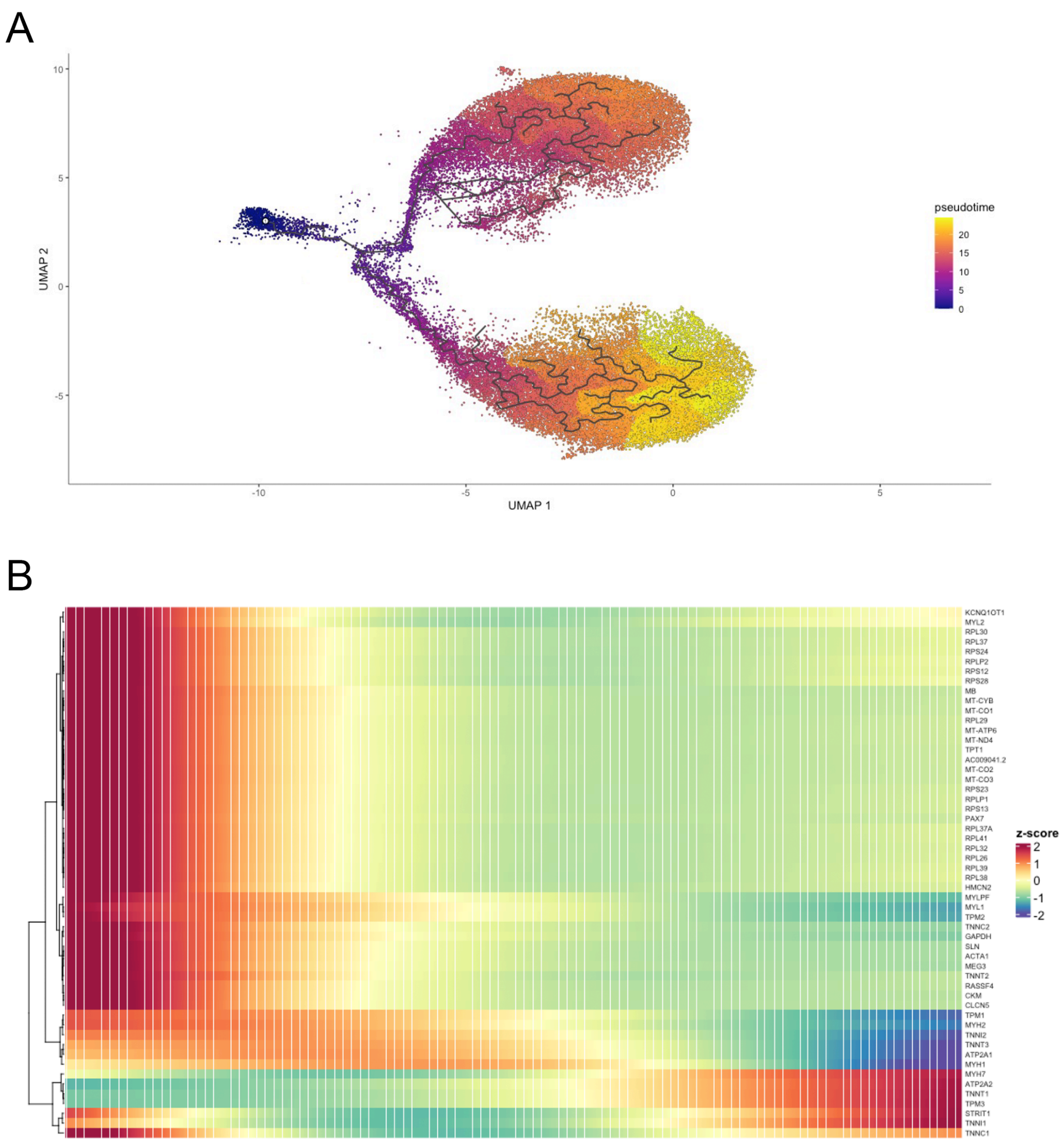

Supplemental Figure 5: Pseudotime trajectories of satellite cells, regenerative and mature nuclei.

A) UMAP showing trajectory of satellite cells, regenerative and mature nuclei of control and DMD samples. B) Heatmap showing the expression of genes during pseudotrajectories.

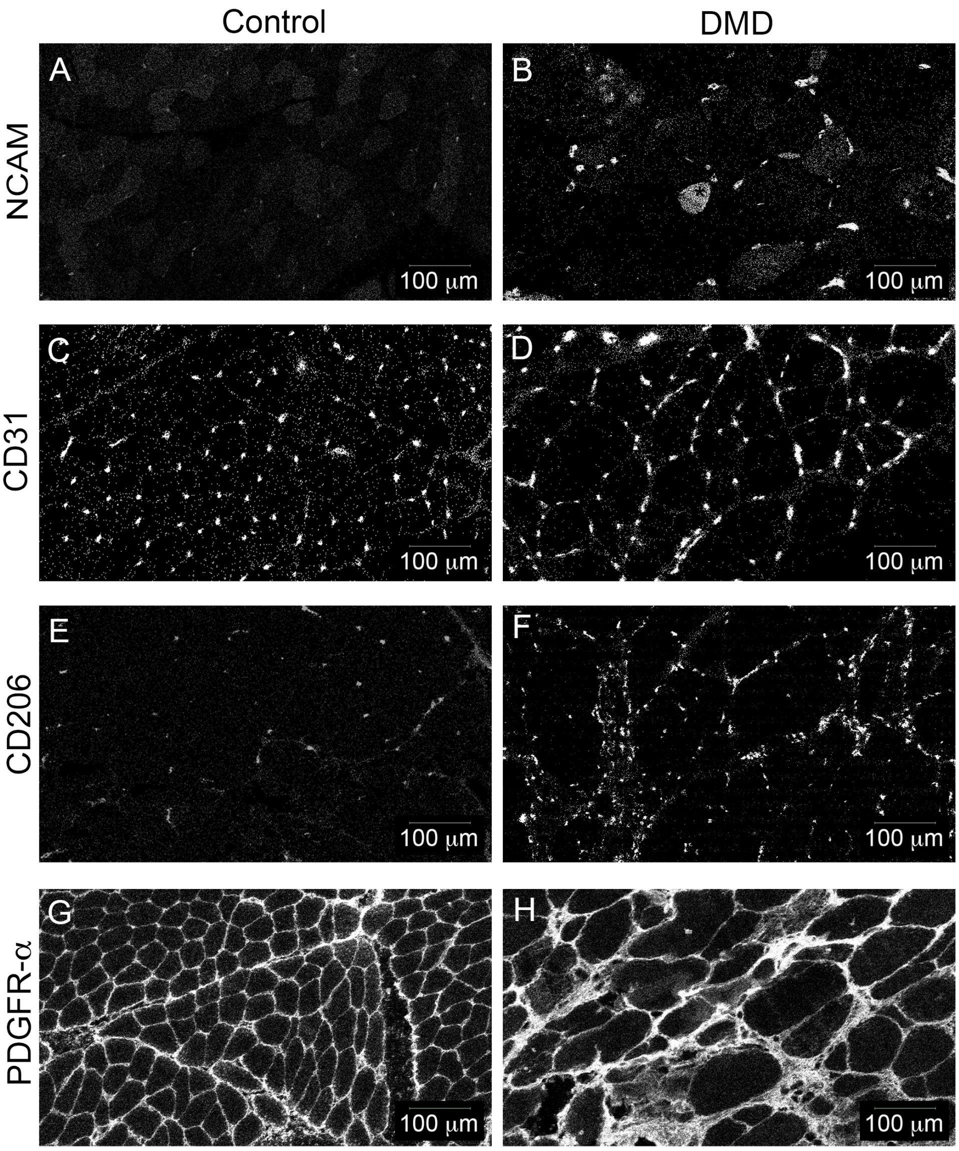

Supplemental Figure 6: Validation of the transcriptomic findings on muscle sections by immunofluorescence.

Control and DMD muscle sections were stained for NCAM, CD31, CD206 and PDGFRα. NCAM was not detected in control samples (A) but an increase of NCAM-positive fibers was observed (B). CD31 staining showed reduction in the number of vessels present in the DMD samples (C and D). We observed an increase in the number of CD206+ macrophages in the DMD samples (F) compared to controls (E). An increase of PDGFRα, a well-known marker of FAP cells, was observed in muscle samples from DMD patients compared to controls (G-H).

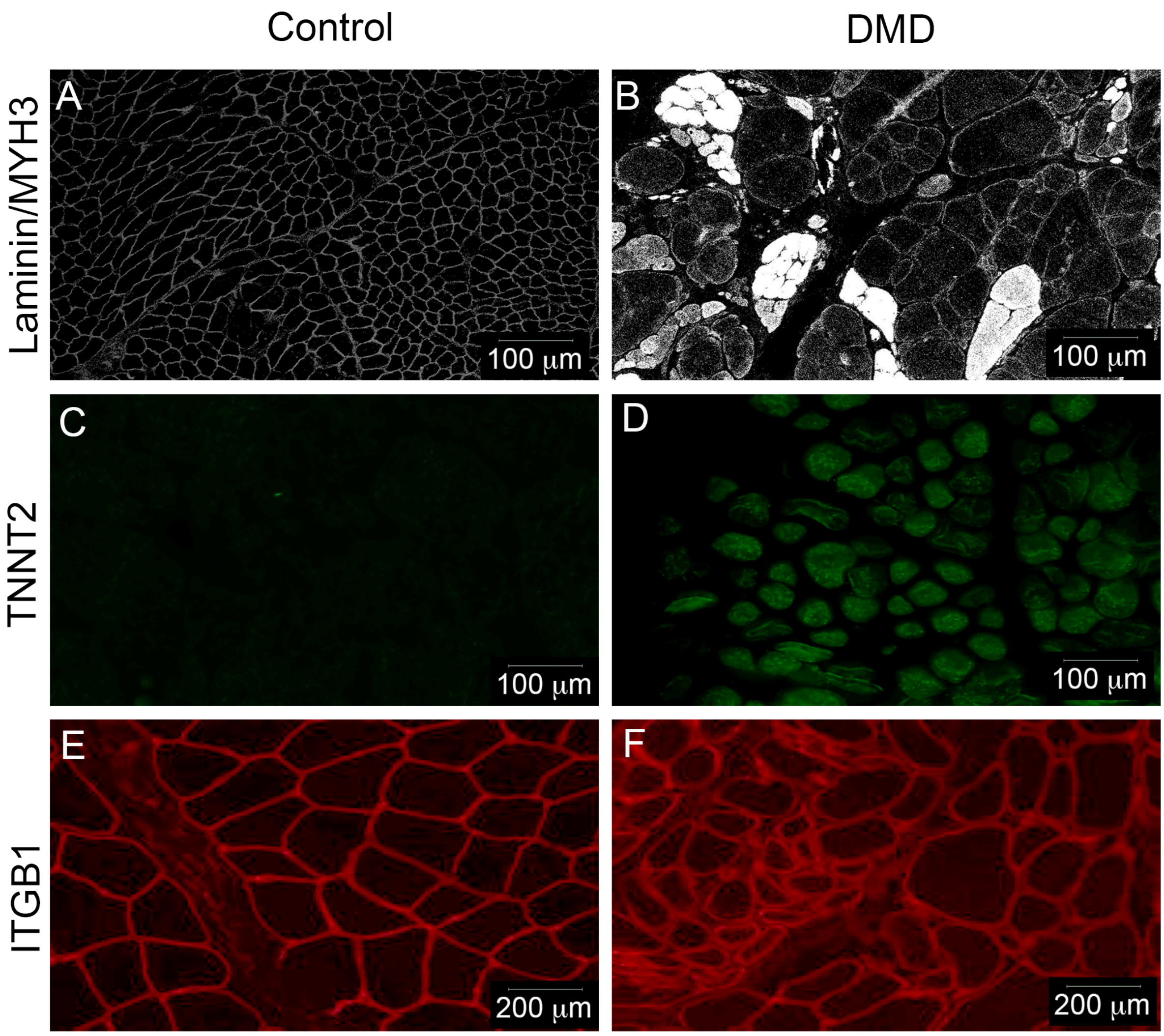

Supplemental Figure 7: Validation of the transcriptomic findings on muscle sections by immunofluorescence.

We studied the expression of some of the proteins encoded by genes upregulated in DMD muscle fibers. We observed an increase in the number of regenerative fibers expressing myosin heavy chain-3 (MYH3) in DMD samples (A-B). Troponin T2 (TNNT2), a protein expressed by cardyomyocites and regenerative fibers was found only in muscle fibers from DMD patients (C-D). Integrin subunit beta-1 (ITGB1) expression was found both in control and DMD samples (G-H).

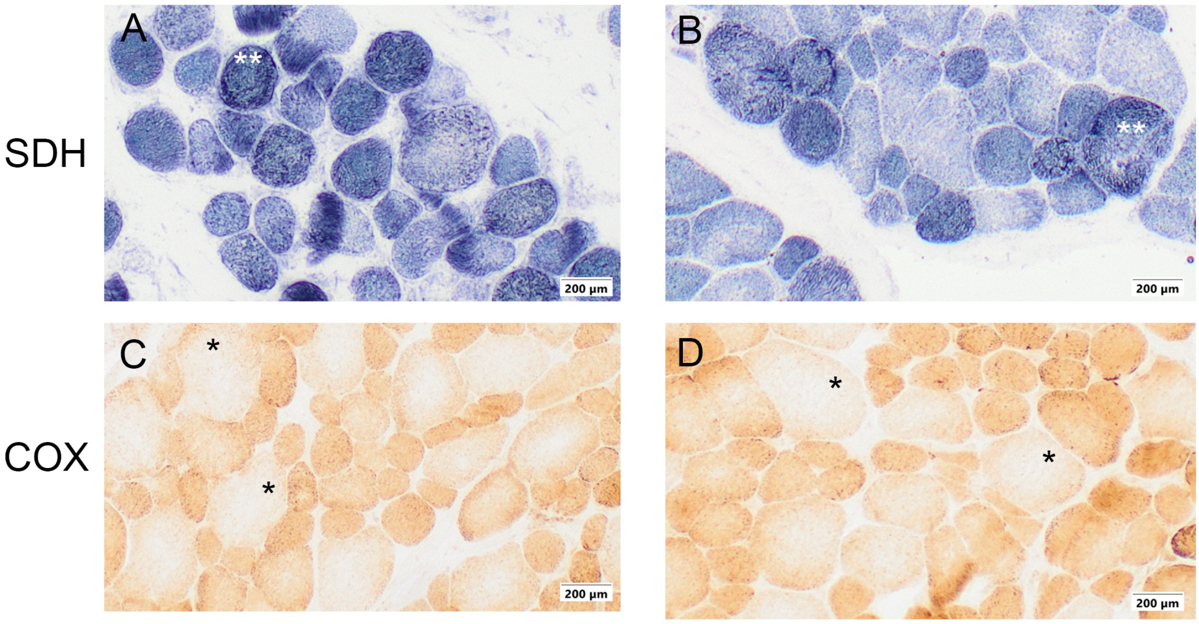

Supplemental Figure 8: Abnormal mitochondrial enzyme expression was found in DMD muscle samples.

We studied the expression of succinate dehydrogenase (SDH) in muscle fibers of patients with DMD and could observed several fibers showing a abnormal subsarcolemmal expression compatible with an accumulation of mitochondria in these fibers (asrerisks in A and B). Cytochrome C-oxidase (COX) staining showed several pale fibers in DMD samples (asterisk) compatible with a hypoactivity of the COX function as was identified in the snRNAseq data (C-D).

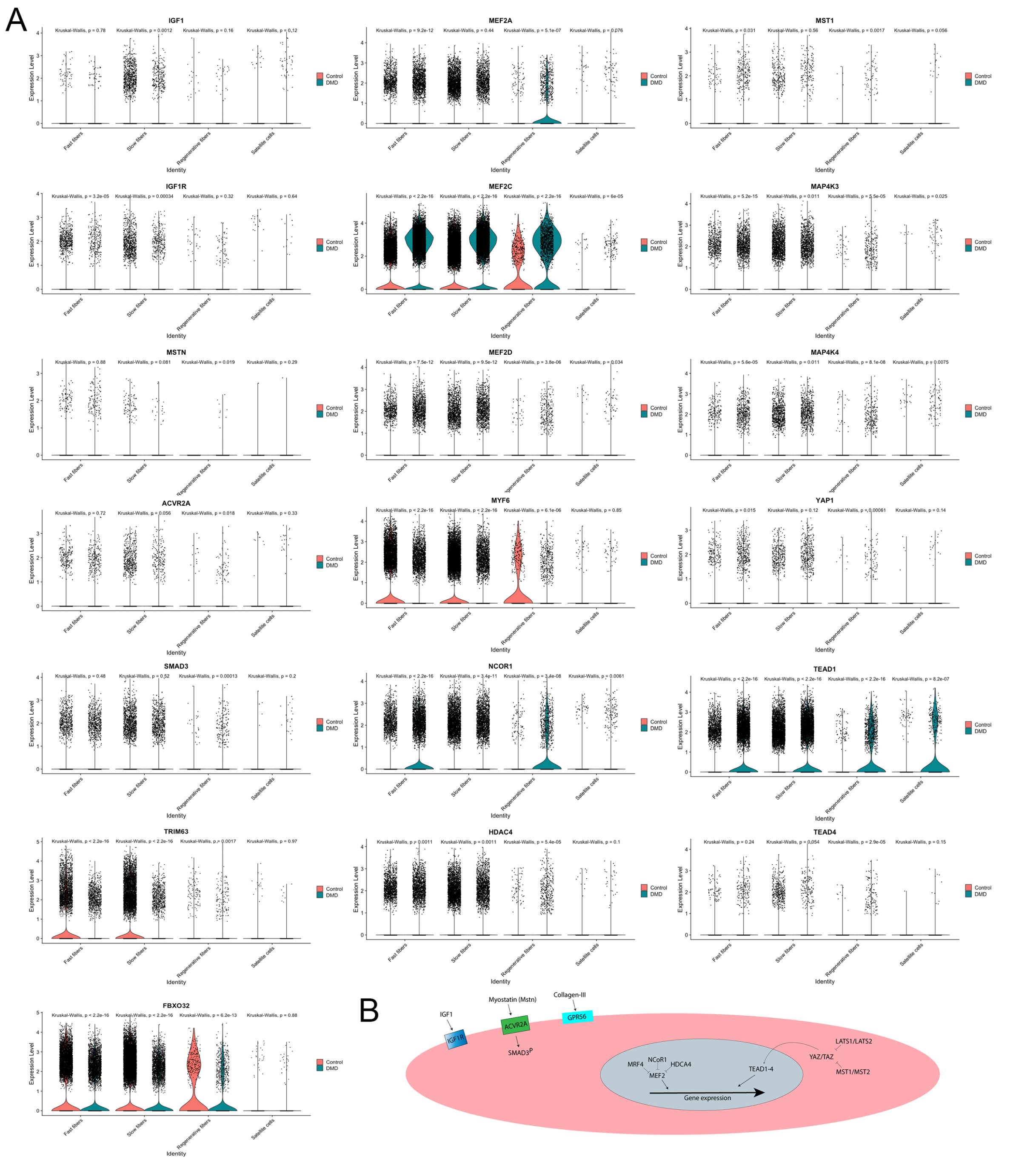

Supplemental Figure 9: Expression of genes related with myogenesis and cell hypertrophy.

A) Violin plot showing the expression of selected genes belonging to specific pathways involved in myogenic program and cell hypertrophy in myonuclei of fast and slow myofibers, regenerative fibers and satellite cells. B) Graph showing selected molecular pathways involved in the myogenic program and cell hypertrophy in skeletal muscle.

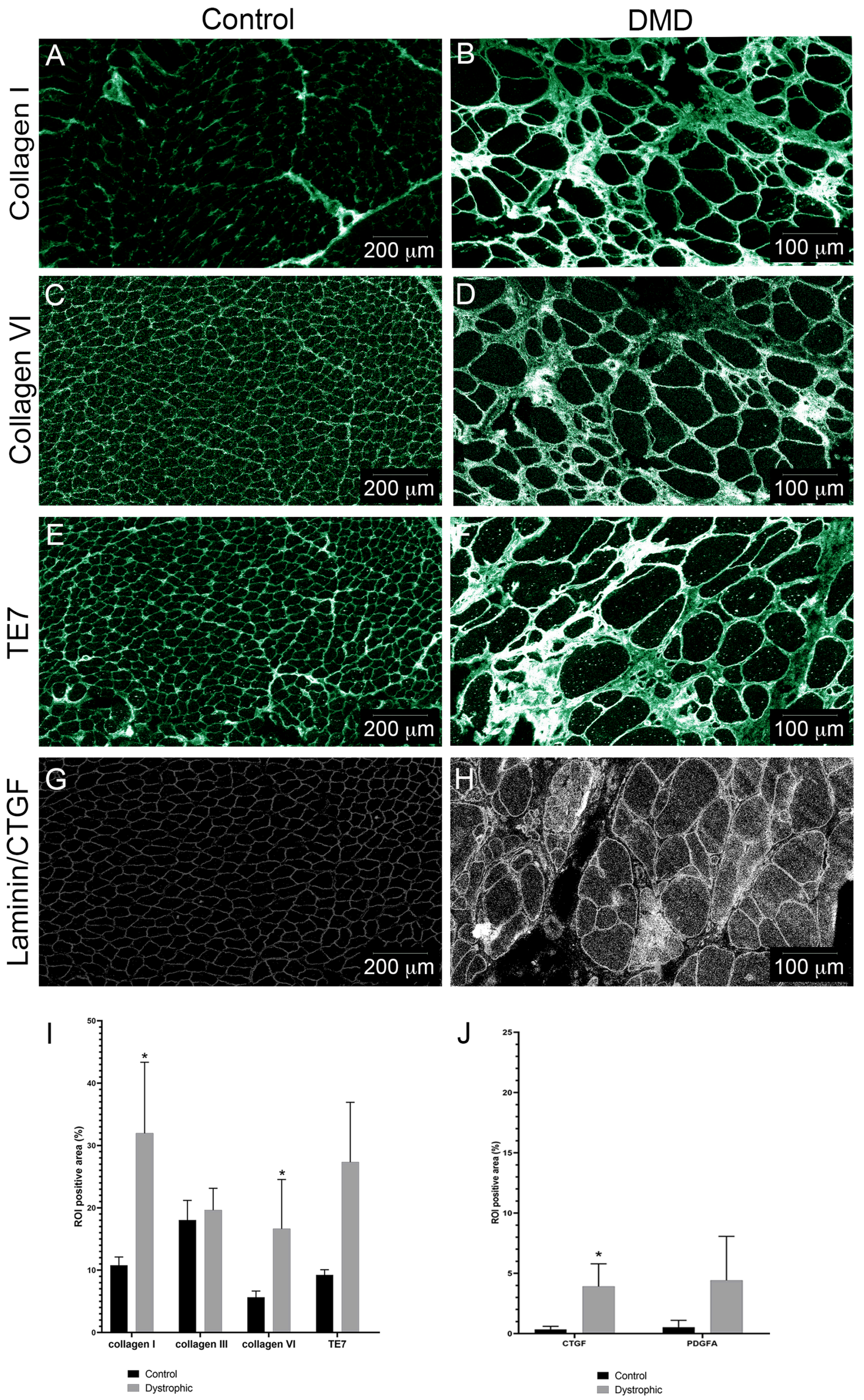

Supplemental Figure 10: Validation of the transcriptomic findings related to fibrosis on muscle sections by immunofluorescence.

We studied the expression of proteins encoded by genes upregulated in fibro-adipogenic progenitor cells (FAPs) in the DMD samples compared to controls. As shown in the panel,

collagen I, collagen VI, TE-7 (a maker of fibroblast) and, connective tissue growth factor (CTGF) expression were increased in DMD samples (B, D, F, H) compared to controls (A, C, E and G). I shows the area of the muscle tissue covered by collagen I, collagen III, collagen VI and TE. We identified significant increase in the area covered by collagen-I and collagen-VI in DMD samples compared to controls (Mann-Whitney U test, p=0.03 and p=0.04 respectively). Bar graph in J shows the area of the muscle covered by CTGF and platelet derived groth factor A (PDGFA). We observed a significant increase in the CTGF area and a trend towards significancy for an increased expression of PDGFA (Mann-Whitney U test, p=0.01 and p=0.06 respectively) in DMD samples compared to controls. Bars show the mean value obtained and errors bars show standard deviation.

	Extracellular matrix components		
Cell type	Upregulated DMD	No change	Upregulated Controls
FAPs	Col1a1<br>Col1a2<br>Col3a1<br>Col5a2<br>Col6a1<br>Col6a3<br>Col6a6<br>Col15a1<br>Col21a1<br>Col27a1<br>Hspg2<br>Lama2<br>Lama4<br>Lamb1<br>Tnxb<br>Cd44<br>Eln<br>Dcn<br>Mfap5	Col4a1<br>Col4a2<br>Col5a1<br>Col6a2<br>Col16a1<br>Col14a1<br>Pcolce<br>Sparc<br>Fn1<br>Itga11<br>Itga8<br>Itgb1<br>Lamb2<br>Lum<br>Cd47<br>Fbln2	Cd36
Regenerative fibers	Itga3<br>Itga7	Col4a3<br>Col4a4<br>Col6a1<br>Hspg2<br>Lama2	Cd36
Mature fibers	Col4a3	Itga7<br>Itgb6	Cd36

Supplemental Figure 11: Expression of genes coding for components of the extracellular matrix by cell type.

Table showing changes in the expression of genes coding for components of the extracellular matrix in control and DMD samples: upregulated in DMD (>0.8 fold increase), not changed (between 0.8 and -0.8 fold increase) and upregulated in controls (>0.8 fold increase).

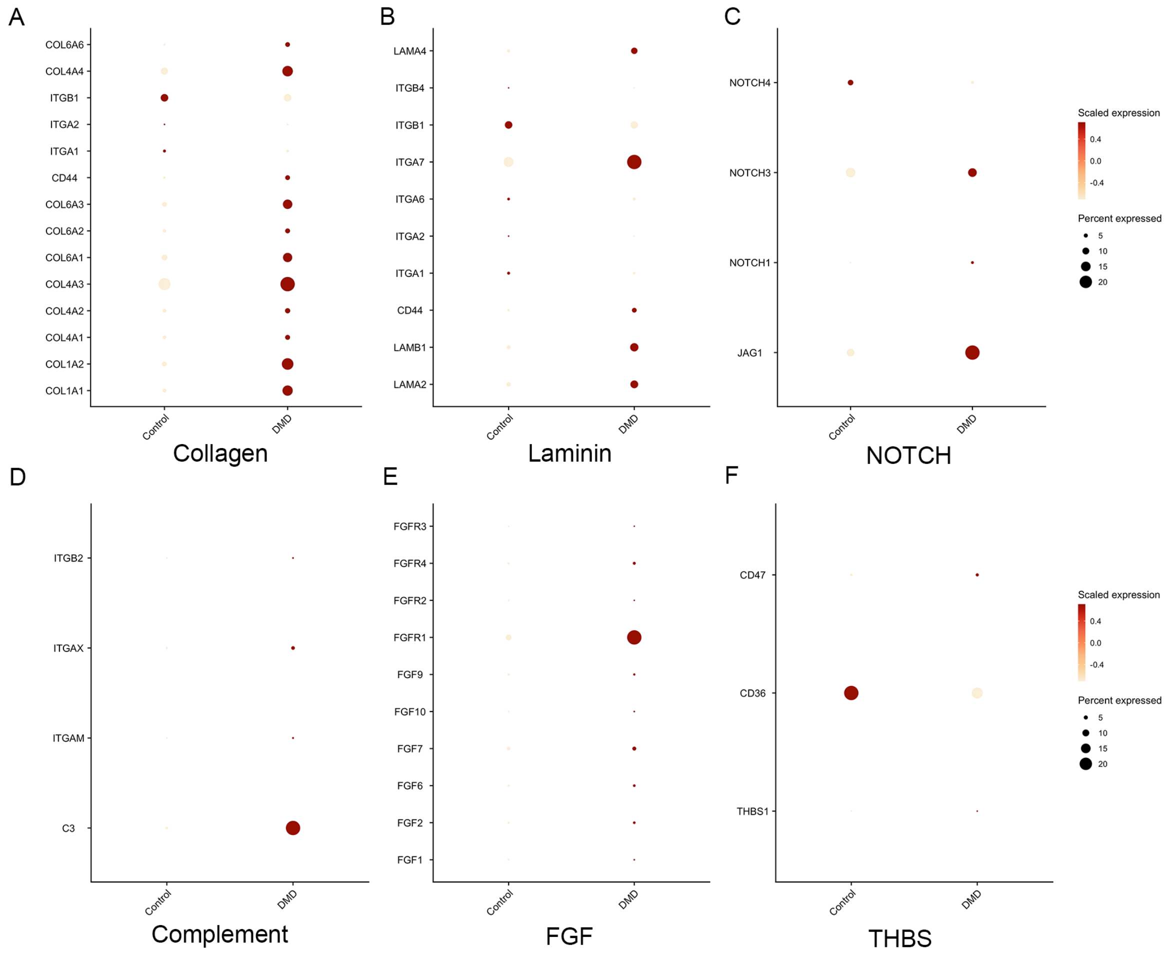

Supplemental Figure 12: Expression levels of ligand-receptor pairs with the highest differences between controls and DMD patients.

We studied the expression levels of genes involved in several molecular pathways shown to be dysregulated in DMD samples compared to controls. Here we are showing the ligand-receptor expression levels of components of the Collagen, Laminin, NOTCH, Complement, FGF and THBS pathways. Scaled expression refers to changes in gene expression comparing DMD and control samples, being the darker the higher the expression. Percent expressed refers to the percentage of nuclei expressing a particular gene, being the bigger the circle the higher the percentage of nuclei expressing that gene.

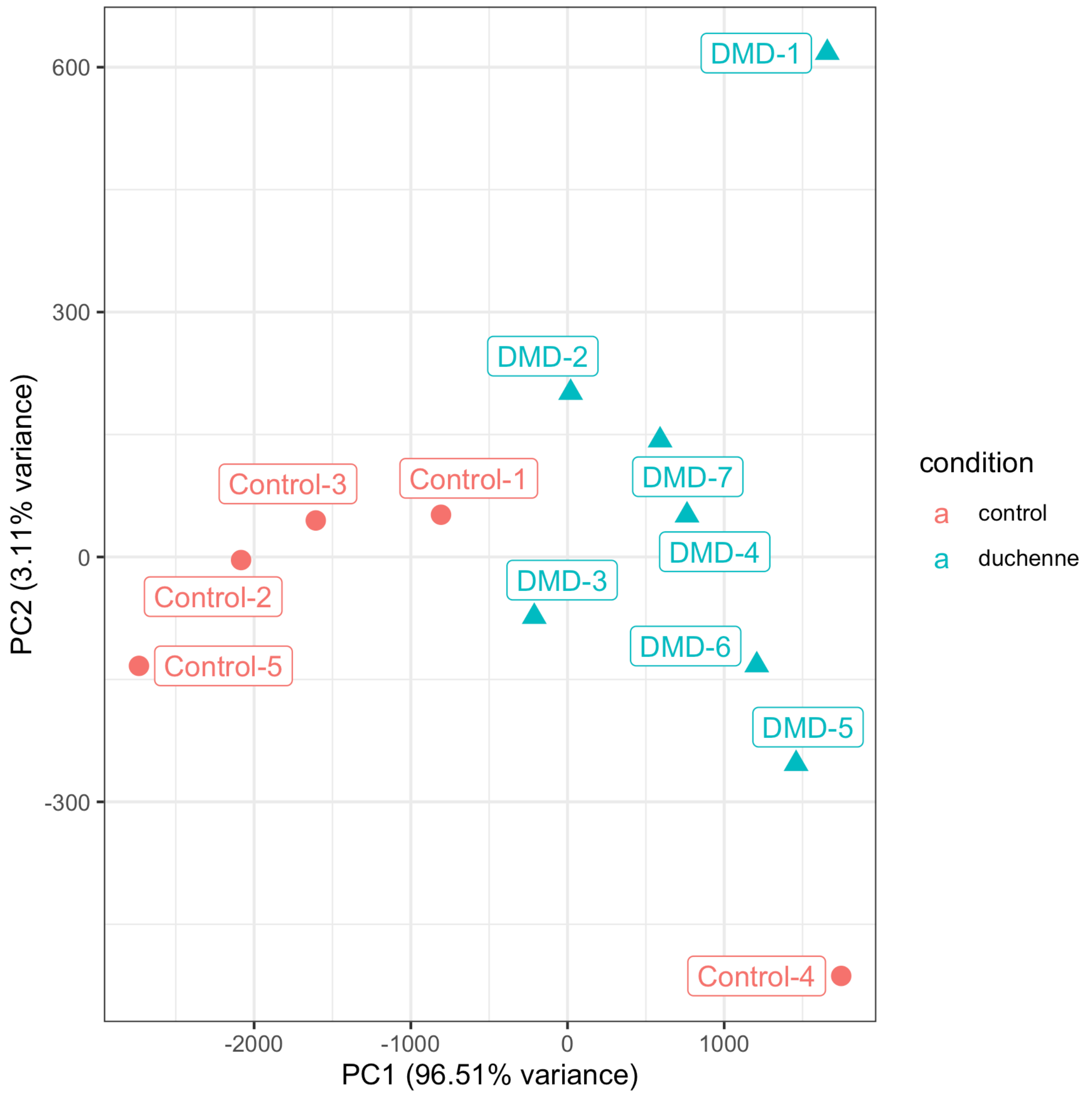

Supplemental Figure 13: PCA analysis of average gene expression of the muscle samples included in the study.
